# Pharmacotherapy guideline concordance for major depressive disorder and its link to functioning via symptom change

**DOI:** 10.1017/cts.2024.562

**Published:** 2024-09-16

**Authors:** Mason T. Breitzig, Fan He, Lan Kong, Guodong Liu, Daniel A. Waschbusch, Jeff D. Yanosky, Duanping Liao, Erika F.H. Saunders

**Affiliations:** 1 Department of Public Health Sciences, Penn State College of Medicine, Hershey, PA, USA; 2 Department of Psychiatry and Behavioral Health, Penn State Health Milton S. Hershey Medical Center, Hershey, PA, USA

**Keywords:** Major depressive disorder, guideline concordance, pharmacotherapy, functioning, symptom severity, patient-reported outcomes

## Abstract

**Introduction:**

Alleviation of symptom severity for major depressive disorder (MDD) is known to be associated with a lagged improvement of functioning. Pharmacotherapy guidelines support algorithms for MDD treatment. However, it is currently unclear whether concordance with guidelines influences functional recovery. A guideline concordance algorithm (GCA-8) was used to measure this pathway in a naturalistic clinical setting.

**Methods:**

Data from 1403 adults (67% female, 84% non-Hispanic/Latino White, mean age of 43 years) with nonpsychotic MDD from the Penn State Psychiatry Clinical Assessment and Rating Evaluation System registry (visits from 02/01/2015 to 04/13/2021) were evaluated. Multivariable linear regression measured associations between GCA-8 and World Health Organization Disability Assessment Schedule 2.0 (WHODAS) scores. Mediation by MDD symptom severity using the Patient Health Questionnaire depression module (PHQ-9) was also evaluated.

**Results:**

This study found a statistically significant improvement in WHODAS scores (functioning) between baseline and final measures (−2 points, *P* < .001) within one year. A one standard deviation increase in the GCA-8 score was associated with a 0.48-point reduction in mean disability score (total effect; *P* = .02) with significant mediation by the change in MDD symptom severity (coefficient = −0.51, *P* < .001) and a nonsignificant natural direct effect of the GCA-8 independent of PHQ-9 change (coefficient = −0.02, *P* = .92).

**Conclusions:**

Higher pharmacotherapy guideline concordance is associated with better functioning for MDD patients; this association likely occurs through improvement in MDD symptom severity rather than directly.

## Introduction

Globally, major depressive disorder (MDD) is recognized as a leading cause of disability and contributes, directly and indirectly, to detriments in work productivity [1–6]. It also impacts daily activities and social and family interaction [7–11]. Prominent somatic MDD symptoms such as fatigue, low energy, disrupted sleep patterns, and change in appetite, as well as their overall severity, may contribute to an individual’s functional decline [6]. However, these symptoms often do not explain all of the variability in the functional impairment associated with MDD [6,8,12]. Regardless of its underlying causes, the detrimental impact of MDD on activities of daily living can be more severe than would be expected for those facing chronic physical disabilities such as diabetes [8,13]. More importantly, evidence shows that these functional impairments linger after remediation of the acute MDD episode [14–16]. The long-term nature of this impact raises essential questions about the applicability of current clinical guidelines and whether their degree of application (guideline concordance) influences functional recovery.

Individuals with MDD can endure functional impairment lasting 425 years after symptom remission [9,10,17]. Consequently, research and the resulting guidelines have made frequent calls to redefine “remission” and incorporate considerations of psychosocial functioning [11,18,19]. However, given the multitude of robust symptom measures, such as the Patient Health Questionnaire depression module (PHQ-9), as well as a heightened interest in symptom remission engendered by outcomes-focused clinical systems (*e.g.*, billing codes in the USA), symptom measures are collected far more frequently than functioning measures [10]. Health systems in the USA are well-equipped to treat acute MDD symptoms for the 40%–66% with MDD who seek care but are less well-equipped to deal with protracted functional impairments [11,17,20].

Research increasingly supports measurement-based care, which promotes frequent assessment of MDD symptoms and functioning [21,22]. Unfortunately, clinical practices are less likely to routinely measure patients’ functioning due to the limited availability of comprehensive but practice-ready (*i.e.*, quick/straightforward for patient use) psychometric measures supported by consensus [10]. Additionally, functional remissions usually occur far beyond the typical one- to two-year treatment window if achieved [8,10,11,23]. Consequently, longitudinal studies on functioning are not always feasible, and psychiatric research often does not track the return to total functional normalcy [8,10,11,23].

Some studies focus on functioning, such as the European Prospective Epidemiological Research on Functioning Outcomes Related to Major depressive disorder (PERFORM) study [8,23]. PERFORM was a two-year observational study of 1159 outpatients from France, Germany, Spain, Sweden, and the United Kingdom, showing that although the functional burden of MDD is intense and spans multiple domains, it is responsive to acute pharmacotherapy [8,23]. However, this improvement tends to be less responsive than MDD symptom improvement and plateaus as symptom measures improve toward subsyndromal levels around the first two years of treatment [8,10]. It has been posited that since subclinical symptoms and perceived residual cognitive symptoms are associated with the risk of recurrence of MDD, management of functional symptoms after clinical remission may be critical to preventing future episodes [10].

The connection between symptom severity for MDD and functioning has been extensively studied. For instance, using longitudinal prospective data from 371 White patients with MDD in the National Institute of Mental Health Collaborative Depression Study, a study found evidence of a linear association between symptom severity and global functioning, emphasizing that higher levels of depressive symptoms may be associated with more severe functional impairments [24].

This finding was later corroborated by a study of 193 Finnish patients, which reported that the two most significant predictors of functioning and social adjustment were the current severity of depression and the cumulative history of depression, each demonstrating significant inverse associations [17]. Although studies demonstrate that treatment of acute episodes can rapidly alleviate some functional impairments, another study showed that the return to functional normalcy occurs more gradually and persists beyond MDD symptom remission, perhaps due to factors beyond depressive symptoms [10,25].

Despite the considerable understanding of the link between symptom severity and functioning and the ability to alleviate functional impairments alongside depressive symptoms, it remains uncertain how specific treatment decisions impact patient functioning [24,25]. Furthermore, it is unclear whether adherence to treatment guidelines (termed guideline concordance) influences such downstream outcomes.

As early as 1995, one study conducted simulations using real-world clinical data and found that guideline-concordant treatment significantly reduced daily role impairment and physical functioning [26]. In a study of the MarketScan database spanning 1992 to 1996, another study similarly found that guideline-concordant practices improved outcomes, namely, extending the time to relapse [27]. However, in the decades since, studies have rarely measured the potential associations between guideline concordance and patient functioning, focusing instead on symptoms [28].

Our Penn State Psychiatry Clinical Assessment and Rating Evaluation System (PCARES) research team and others have developed more specific algorithms to study guideline concordance in practice and, most importantly, its connection to patient outcomes [29–34]. This study applied our previously described guideline concordance algorithm (GCA-8) and examined its association with the World Health Organization Disability Assessment Schedule (WHODAS 2.0), which measures self-reported functioning [32,35]. Our aims were to (1) explore the association between guideline concordance and patient functioning using the GCA-8 and WHODAS and (2) determine whether that association, if any, was mediated by symptom severity as measured using the PHQ-9. We hypothesized that higher concordance with treatment guidelines, through a change (reduction) in MDD symptom severity, would result in better functioning among patients with MDD. Addressing these aims will help enhance the selection of existing treatment strategies and support the implementation of process measures for guideline concordance.

## Methods

### PCARES registry description

The Penn State Psychiatry Clinical Assessment and Rating Evaluation System (PCARES) registry includes a systematic clinical sample of 3556 individuals with mental illness and has been described previously [32,36]. The registry was considered a clinical quality improvement project and thus exempt from review by the Penn State College of Medicine Institutional Review Board (IRB). However, any research project using deidentified PCARES data, including this retrospective study, still necessitates approval by the PCARES steering committee and the Penn State College of Medicine IRB. Accordingly, this study was approved by the PCARES Steering Committee and IRB to use the PCARES data for this research (#00020184) and conducted according to the ethical principles of the Declaration of Helsinki.

### Study participants

Eligible participants (1) were in the PCARES registry (*n* = 3556; individuals with significant cognitive impairment were excluded), (2) had an ICD-10 diagnosis for MDD within one year before and one year after their PCARES encounter (02/01/2015 to 04/13/2021), (3) were 18 years or older at baseline, (4) had at least one PHQ-9 and one World Health Organization Disability Assessment Schedule (WHODAS 2.0) measure (no minimum score required), and (5) had no diagnosis of bipolar disorder or psychosis within one year before baseline. Individuals with significant cognitive impairment were excluded from the registry. After applying the above criteria, we had 1403 eligible participants for whom we calculated a guideline concordance score (described below and previously) and 1241 for complete case analysis [32].

### Guideline concordance algorithm (GCA-8)

Our novel pharmacotherapy guideline concordance algorithm (GCA-8) was informed by CANMAT guidelines and literature on guideline concordance and is described previously [32,28,37]. Eight criteria generate the ordinal score: three focused on prescription sequence (*e.g.*, prescribing a first-line drug before a second-line drug); three on dosing, duration, and modifications; one on drug-drug interactions; and one on visit frequency. According to the algorithm, a point was deducted from the baseline of eight for each failed criterion during the one-year treatment window [32].

### PCARES PRO and EMR data

The PCARES patient-reported outcomes (PRO) battery included, among other questionnaires, the PHQ-9 to measure depression severity and somatic symptoms and the World Health Organization Disability Assessment Schedule (WHODAS 2.0) to measure disability and functioning [12,35,38,39]. For this study, all WHODAS 2.0 scores were tabulated using the 12-item format and the simple scoring strategy, ranging from 0 to 48 (directly comparable to the 12–60 range) as previously described [35,39–41]. PHQ-9 and WHODAS questionnaires with two or more unanswered responses were excluded (described previously) [32]. Questionnaires with one missing value were addressed using mean-imputation (described previously) [32,35,41,42]. Briefly, the WHODAS 2.0 measures six domains of functioning, including cognition, mobility, self-care, getting along (social interaction), life activities (domestic responsibilities, leisure, work, and school), and participation (community activities) [35]. Data from PROs were linked with electronic medical record (EMR) data detailing prescriptions and insurance type, among other practice-specific details described previously [32,36].

In this cross-sectional study (although nested in a retrospective cohort, many of the measures temporally overlap), we used (1) an arithmetic mean of all WHODAS and PHQ-9 scores in one year (except the baseline score; the median number of scores was 4 for each measure), (2) the final WHODAS score in one year (median occurrence at 265 days for both the WHODAS and the PHQ-9), (3) the standard deviation (SD) of WHODAS scores (including baseline) for those with at least three scores (measuring variability in treatment response), (4) and the change in each respective measure from baseline to the final measurement.

### Statistical analyses

Associations between PCARES participants’ WHODAS 2.0 scores and the GCA-8 were evaluated with and without adjustment for the study covariates. Unadjusted analyses evaluated differences in median WHODAS scores across categorical variables (*e.g.*, covariates) via Wilcoxon rank-sum tests and linear associations with continuous variables via simple linear regression (Table 1). Wilcoxon signed-rank test was used to evaluate the median change in WHODAS scores from baseline to the final measure to estimate the one-year improvement for PCARES. Multivariable linear regression analyses were conducted with adjustments for the confounders in Table 1. Finally, an exploratory cross-sectional mediation analysis was conducted for each outcome variable using the “causalmed” (causal mediation) procedure in SAS, which is based on a counterfactual framework [43,44].

**Table 1.** Unadjusted associations between participants’ characteristics, guideline concordance, and other study measures and their baseline WHODAS score (*n* = 1403)

Variable	*n* (%)/mean (SD)	Baseline WHODAS score median (IQR)/β coefficient (SE)	*P* value ^a^
Age (years; 10-year change)	43.06 (17.30)	0.73 (0.16)	< 0.001
Average MDD episode duration (30-day change)	214.72 (120.04)	0.26 (0.07)	< 0.001
Baseline functioning (WHODAS score; range 0–48)	15.7 (10.32)	14 (16)	N/A
Baseline severity (PHQ-9 score; 1-point change)	12.48 (6.83)	0.97 (0.03)	< 0.001
BMI (kg/m^2^)	30.32 (7.98)	0.17 (0.04)	< 0.001
Comorbidity burden (0–11 concurrent disorders)^b^	2.93 (1.95)	1.26 (0.14)	< 0.001
Ethnicity and race			0.002
All others	56 (4)	16 (19)	
Hispanic or Latino	72 (5)	18 (19)	
Non-Hispanic/Latino Black	76 (6)	20 (13)	
Non-Hispanic/Latino White	1172 (85)	14 (16)	
Guideline concordance score (GCA-8)	6.34 (1.31)	−1.25 (0.21)	< 0.001
Scores 0–5 (lowest quartile)	331 (24)	18 (16)	< 0.001
Score 6	352 (25)	15 (16)	
Score 7	447 (32)	13 (15)	
Score 8 (highest concordance)	273 (19)	12 (14)	
Insurance			< 0.001
Commercial	776 (56)	12 (13)	
Medicaid	252 (18)	18 (18)	
Medicare	349 (25)	19 (17)	
Marital status			0.03
Divorced	155 (11)	19 (17)	
Married	577 (41)	14 (17)	
Separated/widowed	86 (6)	17 (18)	
Single	578 (41)	14 (14)	
Recurrent MDD			0.03
Nonrecurrent	615 (44)	13 (16)	
Recurrent	788 (56)	15 (16)	
Rurality			0.08
Rural	193 (14)	15 (15)	
Urban	1190 (86)	14 (16)	
Patient-reported gender			0.06
Female	945 (67)	15 (16)	
Male	458 (33)	14 (15)	
Visit frequency (in 12 months)	21.57 (17.57)	0.13 (0.02)	< 0.001

BMI = body mass index; GCA-8 = guideline concordance algorithm; IQR = interquartile range; MDD = major depressive disorder; PHQ-9 = Patient Health Questionnaire depression module; SD = standard deviation; SE = standard error; WHODAS = World Health Organization Disability Assessment Schedule.

a Testing the null hypothesis that the sample medians are equal (categorical variables) or testing the hypothesis that the regression coefficient in an unadjusted linear model is equal to 0.

b An ordinal measure of the overall burden of comorbidity (including presence or absence of congestive heart failure, stroke, coronary heart disease, diabetes, dyslipidemia, chronic kidney disease, thyroid disorders, hypertension, migraines, fibromyalgia, sleep disorders, anxiety, attention-deficit hyperactivity disorder, trauma, substance use disorder, and obsessive-compulsive disorder).

## Results

### Baseline characteristics of study participants

Unadjusted analyses of the associations between the participants’ characteristics and WHODAS scores for the 1403 participants with MDD in the sample (Table 1) revealed that participants’ degree of self-reported disability (as measured by the baseline WHODAS score) was significantly associated with age (all *P* < .001), where each additional 10 years was associated with a 0.73-point higher WHODAS score, 30 additional days of MDD episode duration (*β* = 0.26; *P* < .001), one-point increase in baseline PHQ-9 score (*β* = 0.97; *P* < .001), one-point increase in body mass index (BMI) (*β* = 0.17; *P*.001), one additional concurrent comorbidity (*β* = 1.26; *P* < .001), and one additional visit (*β* = 0.13; *P* < .001). Conversely, each additional point on the GCA-8 was associated with a significant decrease in a participant’s WHODAS score (*β* = −1.25; *P* < .001).

We also found that the mean (SD) WHODAS score at baseline for the PCARES sample was 15.7 (10.32). Non-Hispanic/Latino Black individuals had the highest median self-reported disability score at baseline 20/48 (Table 1), whereas non-Hispanic/Latino White individuals had the lowest baseline median score (*P* = .002). The median score for those with commercial insurance was 12, while the median for those with either Medicaid or Medicare was 18 and 19, respectively (*P* = .001). Divorced individuals had the highest median disability scores (19; *P* = .03), and those with recurrent MDD had significantly greater self-reported disability than those with nonrecurrent MDD (15 vs 13, *P* = .03). Finally, females reported slightly higher disability with median scores of 15 versus 14 (*P* = .06). There were no significant differences in self-reported disability between individuals who lived in rural versus urban municipalities (*P* = .08).

### WHODAS score distribution at baseline and per the final measure

**Figure 1.**
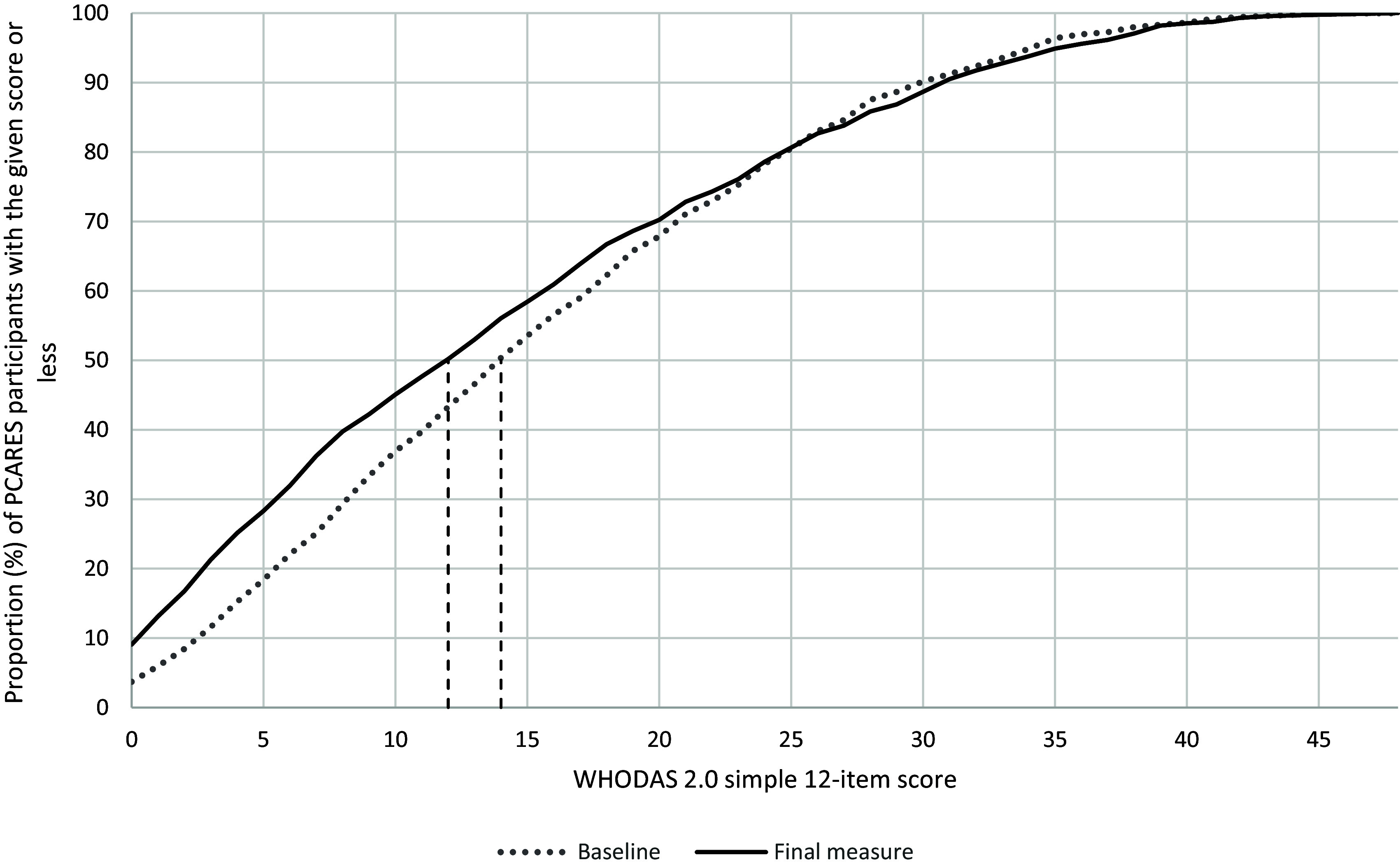
WHODAS scores in the PCARES cohort at baseline per their final WHODAS measurement. The baseline had an *n* = 1241 while the final score had an *n* = 977 due to needing at least two WHODAS measurements; the vertical line represents the 50th percentile score. PCARES = Penn State Psychiatry Clinical Assessment and Rating Evaluation System registry; WHODAS = World Health Organization Disability Assessment Schedule.

The distribution (Figure 1) of self-reported disability was assessed for the PCARES cohort at baseline for 1403 participants with MDD and at their last measure up to one year following baseline (*n* = 977). At baseline, 50% of the PCARES cohort reported having some functional impairment demonstrated by a median score of 14/48. The final measure showed a slight improvement, with the median disability rating reducing to a median score of 12/48. A Wilcoxon signed rank test found that the median change in WHODAS scores from baseline to the final measure was statistically significant (improved) for the 977 individuals who had both a baseline and last follow-up measure within one year of baseline (*P* < .001).

### Adjusted associations between PHQ-9 change and functioning scores

Adjusted associations between PHQ-9 change and each of the three WHODAS outcome score variants (mean, final measure, and SD) were evaluated (Table 2). A one-point increase in PHQ-9 change was significantly associated with lower WHODAS scores. On average, a one-point increase in PHQ-9 change was associated with a 0.62-point decrease in mean WHODAS score (*P* < 0.001), a 0.86-point reduction in the final WHODAS score (*P* < .001), and a 0.09-point reduction in the SD of WHODAS scores (*P* < .001), as expected per literature.

**Table 2.** Adjusted associations between PHQ-9 change in one year and WHODAS scores among PCARES participants with complete data

Outcomes	Sample size^a^	Regression coefficient	Standard error	Coefficient P value	R^2^
Mean WHODAS score in one year (excluding baseline measure)	851	−0.62	0.03	< 0.001	0.78
Final WHODAS score in one year	851	−0.86	0.04	< 0.001	0.77
The standard deviation of 3+ WHODAS scores in one year^b^	688	−0.09	0.02	< 0.001	0.10

The regression coefficient represents slope: mean change in outcome variable per one point increase the change of PHQ-9 score from baseline to the final measure taken for a given individual (the median occurrence of the final measure was at 265 days after baseline); some variables, but not the PHQ-9 change, were standardized before modeling by dividing the variable of interest by the SD of the given predictor as calculated using the entire cohort (n = 1403); the models were adjusted for age, average MDD episode duration, baseline PHQ-9 scores (standardized), baseline WHODAS scores (standardized), BMI, cohort, comorbidity burden (an ordinal score representing the presence or absence of ADHD, anxiety, CKD, CHF, CHD, diabetes, dyslipidemia, fibromyalgia, hypertension, migraines, sleep disorders, stroke, SUD, thyroid disorders, and trauma; observed range: 0–11), insurance type, marital status, visit frequency in 12 months, race and ethnicity, recurrent or nonrecurrent MDD diagnosis at baseline, rural/urban municipal classification, and patient-reported gender; the cohort variable represents the wave of recruitment into the PCARES registry based on the forms that were collected during that period, making it a temporal adjustment.

ADHD = attention-deficit hyperactivity disorder; CHF = congestive heart failure; CHD = coronary heart disease; CVD = cardiovascular disease; MDD = major depressive disorder; PHQ-9 = Patient Health Questionnaire depression module; PCARES = Psychiatry Clinical Assessment and Rating Evaluation System; SD = standard deviation; SUD = substance use disorder; WHODAS = World Health Organization Disability Assessment Schedule.

a The sample size was reduced since 883 individuals had the outcome, but only 958 had the PHQ-9 change variable; thus, the crossover sample size was 851 or less in the case of the WHODAS standard deviation analysis due to needing at least three WHODAS measures.

b Adjusted for the one-year score difference between the baseline WHODAS score and the final measure for a given individual (the median occurrence of the final measure was at 265 days after baseline) rather than the baseline measure.

### Associations between guideline concordance and functioning

To evaluate the potential association between guideline concordance and functioning, a series of unadjusted models were constructed and assessed, each of which identified a significant association between the WHODAS measures and patients’ GCA-8 scores (Table 3), and then the models were adjusted for the covariates listed in Table 1. The adjusted analyses found that the associations between the GCA-8 scores and WHODAS scores remained statistically significant but were attenuated (Table 3). One SD increase in the GCA-8 score was associated with a 0.63-point decrease in the mean WHODAS score in one year (*P* = .004). In comparison, a one SD increase in baseline WHODAS score was associated with a 7.46 increase in the mean one-year WHODAS score (*P* < .001). Similarly, a one SD increase in GCA-8 was associated with a 0.83-point decrease in the final WHODAS score (*P* = .002). Finally, a one SD increase in GCA-8 was associated with a 0.29-point reduction in the intraindividual SD of WHODAS scores up to one year after the initial encounter (*P* = .002).

**Table 3.** Associations between standardized scores of guideline concordance and patient-reported functional disability scores

Outcomes	Model	Predictor of interest	n	Regression coefficient	SE	Coefficient P value	R^2^
Mean WHODAS score in one year (excluding baseline measure)	Unadjusted	GCA-8	883	−2.22	0.35	< 0.001	0.04
	Adjusted	GCA-8		−0.63	0.22	0.004	0.68
		Baseline WHODAS		7.46	0.30	< 0.001	
Final WHODAS score in one year	Unadjusted	GCA-8	883	−2.41	0.38	< 0.001	0.04
	Adjusted	GCA-8		−0.83	0.26	0.001	0.61
		Baseline WHODAS		7.48	0.35	< 0.001	
The standard deviation of 3+ WHODAS scores in one year^a^	Unadjusted	GCA-8	693	−0.35	0.09	< 0.001	0.02
	Adjusted	GCA-8		−0.29	0.09	0.002	0.08

Regression coefficient represents slope; mean change in outcome variable per one SD increase in guideline concordance score; scores were standardized before modeling by dividing the variable of interest by the SD of the given predictor as calculated using the entire cohort (*n* = 1403); models also adjusted for age, average MDD episode duration, baseline PHQ-9 scores, BMI, cohort, comorbidity burden, race and ethnicity, insurance type, marital status, visit frequency in 12 months, recurrent or nonrecurrent MDD diagnosis at baseline, rural/urban classification, and patient-reported gender; the cohort variable represents the wave of recruitment into the PCARES registry based on the forms that were collected during that period, effectively making it a temporal adjustment. The adjusted model here does not contain the sample size-limiting variable representing PHQ-9 change; thus, the effect differs slightly from the total effect in Table 4.

BMI = body mass index; GCA-8 = guideline concordance algorithm; MDD = major depressive disorder; SD = standard deviation; SE = standard error; PCARES = Psychiatry Clinical Assessment and Rating Evaluation System; WHODAS = World Health Organization Disability Assessment Schedule.

a Adjusted for the one-year score difference between the baseline WHODAS score, the final measure for a given individual, rather than the baseline measure.

**Table 4.** Mediation analysis examining the pathway between guideline concordance score and WHODAS score via PHQ-9-measured symptom severity (*n* = 851)

Outcomes	Effect	Regression coefficient	SE	Coefficient P value
Mean WHODAS score in one year (excluding baseline measure)	Total effect	−0.53	0.22	0.01
	PHQ-9-mediated effect	−0.51	0.13	< 0.001
	Remaining (natural direct) effect	−0.02	0.18	0.92
Final WHODAS score in one year	Total effect	−0.71	0.26	0.005
	PHQ-9-mediated effect	−0.70	0.17	<0.001
	Remaining (natural direct) effect	−0.02	0.20	0.92
One-year change in the WHODAS score	Total effect	0.59	0.26	0.03
	PHQ-9-mediated effect	0.68	0.16	< 0.001
	Remaining (natural direct) effect	−0.09	0.22	0.71

The regression coefficient represents the mean change in the outcome variable per one standard deviation increase in the guideline concordance score; models also adjusted for age, average MDD episode duration, baseline PHQ-9 scores, BMI, cohort, comorbidity burden (described earlier), insurance type, marital status, visit frequency in 12 months, race and ethnicity, recurrent or nonrecurrent MDD diagnosis at baseline, rural/urban municipal classification, and patient-reported gender; the models examining the outcome of mean WHODAS and final WHODAS score in one year were also adjusted for the standardized baseline WHODAS score.

BMI = body mass index; GCA-8 = guideline concordance algorithm; MDD = major depressive disorder; PHQ-9 = Patient Health Questionnaire depression module; SE = standard error; WHODAS = World Health Organization Disability Assessment Schedule.

### Cross-sectional mediation involving the GCA-8, symptom severity, and functioning

In a final analytic step, we evaluated the potential mediation of the association between guideline concordance and functioning by a change in symptom severity. An adjusted mediation analysis was conducted for each version of the WHODAS metric, with the change in PHQ-9 scores as the mediator between the GCA-8 and the WHODAS scores. For every SD increase in the GCA-8 score, the mean WHODAS score decreased by 0.53 points (*P* = .02; Table 4). This total effect was decomposed into a significant effect mediated by the change in the PHQ-9 score of −0.51 (*P* < .001) and a nonsignificant natural direct effect of −0.02 (*P* = .92), where the PHQ-9 change does not explain the influence of GCA-8. In this case, the mediator pathway could be entirely responsible for the association between a patient’s GCA-8 score and their mean WHODAS score in one year. While the GCA-8 score increases, it is possible that the difference between baseline PHQ-9 and final PHQ-9 also increases, which is associated with a lower mean WHODAS score.

For the final WHODAS score, a one SD increase in the GCA-8 score was associated with a 0.72-point decrease in patients’ final WHODAS score in one year (*P* = .005). However, just as above, the natural direct path was not statistically significant (*P* = .92), and the indirect path demonstrated that PHQ-9 change drove the total effect (*P* < .001). Lastly, the association with the one-year change in WHODAS was examined. A one SD increase for the GCA-8 was found to be associated with a 0.59 increase in WHODAS change (*P* = .03). The indirect mediated effect was again statistically significant (*P* < .001). In contrast, the direct, PHQ-9-independent GCA-8 effect was not statistically significant (*P* = .71).

## Discussion

This study examined the association between guideline concordance (via GCA-8 scores) for MDD treatment and patient functioning and whether alleviating MDD symptoms could mediate this relationship. After adjusting for standard sociodemographic covariates, as well as practice-specific covariates (*e.g.*, average MDD episode duration), the results demonstrated that (1) higher guideline concordance scores were associated with significantly lower WHODAS scores, and (2) this significant association could be explained by the change (improvement) in depression symptom severity over time. This study contributes to the literature by showing that the degree of guideline concordance for a given patient’s pharmacotherapy is essential, such that more guideline-concordant pharmacotherapy is associated with reduced symptom severity and, perhaps consequently, lower functional impairment.

Even though studies have established the association between symptom severity and functioning, including the seminal 12-month naturalistic Sequenced Treatment Alternatives to Relieve Depression (STAR*D), as reaffirmed here (Table 2), this study is among the first to report an association between guideline concordance and patient-reported functioning (Table 3) in a modern clinical cohort [10,23,24,45,46]. In 1999, a study provided evidence of a linear association between guideline concordance and reduction in functional limitations [26]. In 2006, the STAR*D study showed that remission is more likely for those with better baseline functioning (via measurement of physical and mental functioning with the 12-item Short Form Health Survey) [46,47]. However, per design, all patients received guideline-concordant care and, like many other studies, did not provide information on the role of guideline concordance [46,47].

Much later, a similar – but more functioning-focused – study in 2018, PERFORM, measured the “switch of antidepressant” and found that switching for the first time was associated with a significantly higher disability score on the Sheehan disability scale (SDS) [8]. Lastly, in 2020, another study showed that the time spent on medication was marginally associated with lower odds (95% CI: 0.83, 0.99) of having a social impairment [45]. This study expands the literature by measuring more domains of concordance in a population receiving modern pharmacotherapies. More rigorous studies are needed to elucidate causality between guideline-concordant treatment and functioning.

To further describe the cross-sectional pathway between guideline concordance and WHODAS scores that we observed, this study also evaluated the change in PHQ-9 score as a mediator. We previously demonstrated that the GCA-8 score correlates with participants’ PHQ-9 scores and that avoiding one discordant event was associated with scoring approximately one point lower on PHQ-9 – a clinically relevant improvement [32]. Additionally, the results in Table 2 and existing evidence corroborate that a reduction in PHQ-9 scores via treatment is significantly associated with functional remediation [10,23,24,45]. This overlap could be due to changes in the somatic symptoms of fatigue, energy loss, sleep disruption, change in appetite, and overall symptom severity, measured in the PHQ-9, as mentioned above [6,12,42]. Given this information, cross-sectional mediation models were constructed to determine whether the association between guideline concordance and functioning could be wholly or partly attributable to an improvement in symptom severity.

Results showed that PHQ-9 scores may fully mediate the association between GCA-8 and WHODAS scores (Table 4). Moreover, while the total effect of each analysis was statistically significant, all three of the PHQ-9-independent GCA-8 effects were nonsignificant (Table 4). In other words, lower symptom severity fully accounted for better functioning scores associated with higher guideline concordance. These results indicate that, among patients presenting for treatment of depression, a higher GCA-8 score (*i.e.*, better concordance with treatment guidelines) may translate to significant improvement in both depression symptom severity and functioning [6,8,10,23,24]. Future longitudinal work should consider that some drugs and doses influence patients’ functioning through several mechanisms and that the treatment strategies supported by clinical guidelines can have a lesser negative impact on patient functioning, a more significant effect on improving functioning, or both. Guideline concordance may also directly impact aspects of functioning that are not measured by the WHODAS and may be independent or upstream of symptom severity, such as cognitive functioning [23].

In addition to these analyses, this study helps characterize disability in PCARES, which connects to other clinical populations with MDD. The data show that the patient-reported degree of disability significantly varied by most patient characteristics, including age, BMI, ethnicity, insurance type, and marital status. However, it did not differ significantly by rurality or patient-reported gender. Several studies find similar but inconsistent results for age, ethnicity, sex, and marital status [8,15,17,39,45]. The results of this study also showed that non-Hispanic/Latino Black or Hispanic/Latino individuals had significantly greater median disability scores, often attributed to disparities in socioeconomic status, education, and health care [48,49].

This study had several strengths, such as involving a large sample of clinically diverse (*e.g.*, different comorbidities and insurance types) participants who received an array of pharmacotherapeutics in a natural clinical setting. An additional strength is our ability to evaluate patient outcomes in multiple ways due to having repeated measures of symptom severity (PHQ-9) and functioning (WHODAS). This study also had several limitations. Although we had some temporality in assessments where we evaluated the baseline measure of disability versus the final measure, the temporal overlap of the GCA-8 with our outcome measures precludes the establishment of causality. The PCARES sample includes those who seek treatment in a specific geographic region and, as such, has limited generalizability to the rest of the USA. There was no information on treatment adherence, psychotherapy, providers, tobacco smoking, treatment received *out of network*, or treatment received before 2015; such residual confounding may impact score distributions and further limit generalizability. As not all PCARES participants were at the same stage in their treatment, and not all patients received the same number of follow-up rating measures, the study may over- or underestimate the association between the GCA-8 and WHODAS. Loss to follow-up of those who ceased care due to improvement or nonresponse may also bias these associations toward or away from the null. This limitation is somewhat mitigated by looking at the associations only among those with multiple measures. Finally, diagnostic errors, lack of individual clinical context, and lack of information on treatment adherence or providers may lead to some outcome misclassification and mistakes in measuring guideline concordance.

The results of our study suggest that concordance with pharmacotherapy guidelines for MDD treatment is significantly associated with patient-reported functioning, such that higher treatment concordance (higher GCA-8 scores) is associated with better functioning scores. We showed that this relationship may be entirely due to a change in patients’ reported symptom severity, assuming the proposed causal pathway exists. We also demonstrated that greater guideline concordance is associated with better patient outcomes after adjustment for sociodemographic characteristics and clinical metrics. Future work should elucidate the causal relationship between guideline concordance and patient outcomes in real-world scenarios to improve process quality control and MDD treatment outcomes.

## Data Availability

The data supporting this study’s findings are available from the corresponding author upon reasonable request.
